# P-1618. Real-world Evaluation of Multiplex Pneumonia Panel Performance and its impact on Antibiotic Utilization

**DOI:** 10.1093/ofid/ofae631.1785

**Published:** 2025-01-29

**Authors:** Neeraja Swaminathan, Hannah Imlay, Ali Earl, Karen Fong, Emily S Spivak

**Affiliations:** University of Utah, Salt Lake City, Utah; University of Utah Health, Salt Lake City, UT; University of Utah, Salt Lake City, Utah; University of Utah, Salt Lake City, Utah; University of Utah School of Medicine, Salt Lake City, Utah

## Abstract

**Background:**

Gaps exist in understanding how molecular syndromic testing aligns with culture-based diagnostics and if clinicians use this information to optimize antimicrobial use. The Biofire FilmArray® Pneumonia panel (PN-panel) includes 15 bacterial, 3 atypical, and 8 viral targets as well as antimicrobial resistance genes. We assessed performance and utilization of this panel in the absence of antimicrobial stewardship program (ASP) oversight post-implementation.

**Figure 1:**
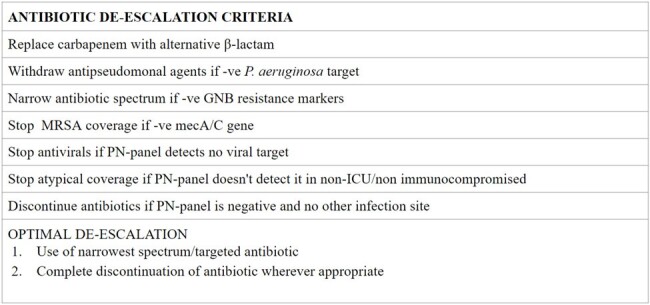
Antibiotic de-escalation criteria

**Methods:**

We audited 79 specimens (49 bronchoalveolar lavage & 30 endotracheal aspirates) from 67 patients (August 1, 2023, to November 30, 2023). Demographics, pneumonia panel testing, and concurrent respiratory culture results were recorded. Concordance between PN-panel and culture was defined as identical organisms or dominant organism matching. Instances where culture showed normal oral flora/no growth while PN-panel was negative were also considered concordant. We categorized pre- and post- PN-panel antibiotic selection as escalation, no change, or degrees of de-escalation (defined in Figure 1). De-escalation was evaluated between three infectious disease clinicians to reach consensus.

**Figure 2:**
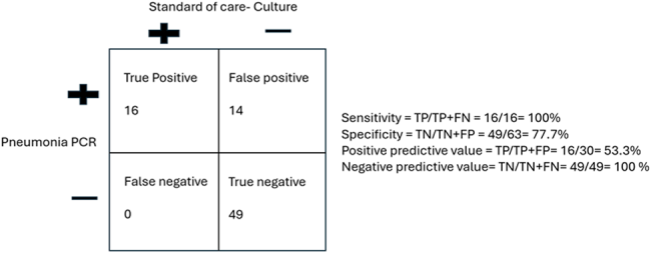
Performance of PN-panel compared to culture-based diagnostics

**Results:**

The mean age was 57 years, with 67% male and 34% female. PN-panel testing was positive in 30/79 patients including 11 polymicrobial specimens, 5 with viral targets and 5 with resistance gene detections. 20.3% (16/79) of specimens had positive cultures. Concordance of PN-panel and culture results was 86.1% (68/79) with PN-panel demonstrating a PPV of 53.3% and an NPV of 100% (Figure 2). Analysis of antibiotic prescribing post- PN-panel was done separately for immunocompromised and non-immunocompromised cohorts (Table 1), revealing significant opportunity for de-escalation in both groups, particularly prominent in the non-immunocompromised subset. Even after exclusion of patients with other microbiologically confirmed infections, only 41.6% of eligible non-immunocompromised patients de-escalated antibiotics.

**Table 1:**
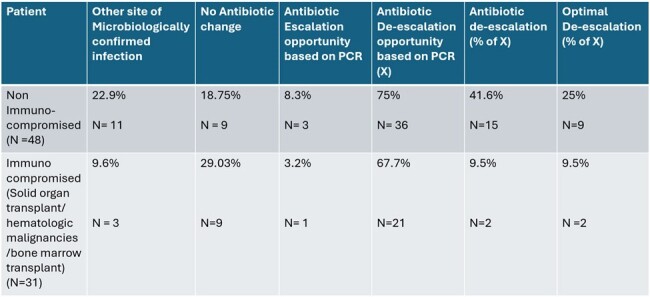
Analysis of post PN-panel Antibiotic prescribing

**Conclusion:**

Despite the PN-panel’s high sensitivity and NPV, antibiotic de-escalation occurred only in a minority of patients. Collaboration with ASP teams is likely to enhance its clinical utility by guiding result interpretation and antibiotic management.

**Disclosures:**

**All Authors**: No reported disclosures

